# Macrophage pyroptosis and its crucial role in ALI/ARDS

**DOI:** 10.3389/fimmu.2025.1530849

**Published:** 2025-02-14

**Authors:** Yuju Cai, Luorui Shang, Fangyuan Zhou, Mengqi Zhang, Jinxiao Li, Shuhan Wang, Qifeng Lin, Jianghua Huang, Shenglan Yang

**Affiliations:** ^1^ Department of Clinical Nutrition, Union Hospital, Tongji Medical College, Huazhong University of Science and Technology, Wuhan, Hubei, China; ^2^ Cancer Center, Union Hospital, Tongji Medical College, Huazhong University of Science and Technology, Wuhan, China

**Keywords:** ALI (acute lung injury), ARDS (acute respiratory distress syndrome), pyroptosis, macrophage - cell, treatment

## Abstract

Acute lung injury(ALI)/acute respiratory distress syndrome(ARDS) is a severe clinical syndrome characterized by high morbidity and mortality, primarily due to lung injury. However, the pathogenesis of ALI/ARDS remains a complex issue. In recent years, the role of macrophage pyroptosis in lung injury has garnered extensive attention worldwide. This paper reviews the mechanism of macrophage pyroptosis, discusses its role in ALI/ARDS, and introduces several drugs and intervening measures that can regulate macrophage pyroptosis to influence the progression of ALI/ARDS. By doing so, we aim to enhance the understanding of the mechanism of macrophage pyroptosis in ALI/ARDS and provide novel insights for its treatment.

## 1 Introduction

ALI/ARDS is a common respiratory disease, which is an acute hypoxic respiratory insufficiency caused directly or indirectly by various intrapulmonary or extrapulmonary factors, with progressive hypoxemia and respiratory distress as the main clinical feature (1, 2). About 3 million people suffer from ALI/ARDS around the world every year, and the mortality is as high as 35%-46% (3). The risk factors for ALI/ARDS include direct and indirect lung injuries. The direct factors include infectious pneumonia, aspiration of stomach contents and severe trauma. On the contrary, indirect factors arise from a systemic inflammatory response triggered outside the lungs, such as sepsis from non-pulmonary infections, non-thoracic trauma, pancreatitis, severe burns, blood product transfusion, and reperfusion edema following lung transplantation or thrombectomy (4). Currently, no effective treatment exists to reduce mortality or improve the prognosis of patients with ALI/ARDS. Therefore, exploring the pathogenesis of ALI/ARDS is crucial for developing effective treatments.

The pulmonary innate immune system serves as the first line of defense against external stimuli by recognizing pathogen-associated molecular patterns (PAMPs) and damage-associated molecular patterns (DAMPs). Innate immune cells located in the lung epithelium play an essential role by producing pro-inflammatory factors to eliminate pathogens and releasing anti-inflammatory factors to maintain lung homeostasis (5). Pulmonary macrophage is a critical cell of the pneumonic innate immune system, expressing pattern recognition receptors(PRRs) to recognize PAMPs and DAMPs (6). Once the PAMPs or DAMPs are recognized by PRRs, macrophages will initiate a series of immune responses, including inflammasome activation and release of inflammatory cytokines. These processes, in addition to removing pathogens, also lead to damage and pyroptosis of macrophages. On the one hand, activated inflammasomes recruit and activate inflammatory caspases and subsequently lead to activation and release of pro-inflammatory factors like IL-1β and IL-18, which further aggravates the inflammatory response (7). On the other hand, activated caspases can also cleave and activate gasdermin protein, contributing to cell membrane perforation and cell swelling, eventually inducing cell pyroptosis (8). Generally, macrophage pyroptosis contributes to the occurrence and development of ALI/ARDS. However, the precise regulatory mechanisms of macrophage pyroptosis remain incompletely understood and involve a range of signaling pathways and other regulatory networks (9, 10). The regulation of macrophage pyroptosis may represent a novel therapeutic direction for ALI/ARDS. In this review, we summarize the known mechanisms of macrophage pyroptosis and its impact on ALI/ARDS.

## 2 ALI/ARDS

**Figure 1:**
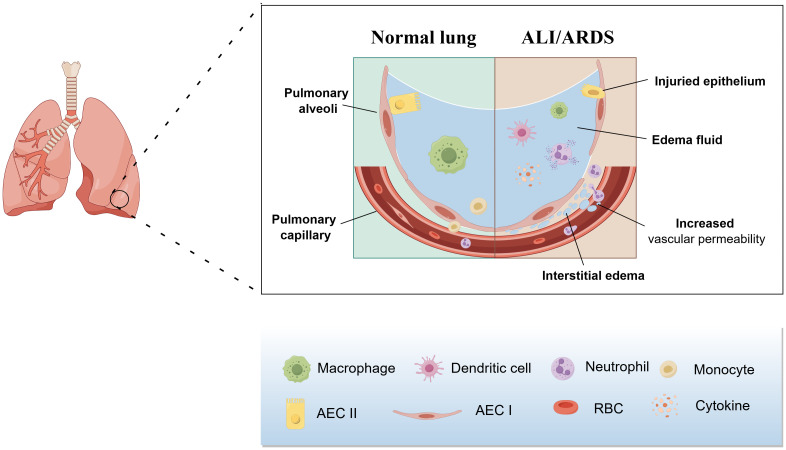
Pathological features of ALI/ARDS. The pathologic features of ALI/ARDS include diffuse necrosis of alveolar capillary endothelial cells and epithelial cells, increased permeability of the pulmonary capillary endothelial and alveolar epithelial barriers, accumulation of protein-rich edema fluid, extensive pulmonary hemorrhage, and thrombosis in the alveolar hyaloid membrane and capillaries.

Ashbaugh, with his co-workers, first put forward the term ALI/ARDS in 1967 (11). In 1994, American-European Consensus Conference(AECC)proposed the clinical definition of ALI/ARDS: rapid onset of respiratory failure(ALI is diagnosed when 200 mmHg<PaO_2_/FiO_2_<300 mmHg and the criteria for ARDS are met when PaO_2_/FiO_2_< 200mmHg); chest radiography showing bilateral diffuse pulmonary infiltration; absence of increased pulmonary artery wedge pressure; and no clinical manifestation of left atrial hypertension (12, 13). Subsequently, the ARDS was classified into the following three categories based on the degree of hypoxemia according to the later Berlin definition: mild ARDS(PaO_2_/FiO_2_ = 200-300mmHg); moderate ARDS(PaO_2_/FiO_2_ = 100~200mmHg); and severe ARDS(PaO_2_/FiO_2_<100mmHg) (14). Although the background of onset is different between ALI and ARDS, the clinical symptoms, pathophysiological features and specific targets for pharmacological interventions are similar, which contributes to the fact that ALI and ARDS are usually studied as a whole (15). The pathological characteristics of ALI/ARDS include depressed pulmonary compliance, increased pulmonary vascular permeability and alveolar and interstitial pulmonary edema (**Figure 1**) (16, 17), while the main clinical symptoms are refractory hypoxemia and progressive exacerbation of hypoxic respiratory failure (1).

ALI/ARDS is characterized by life-threatening lung injury, in which immune mechanisms play an essential role. In response to lung damage, the immune system initiates a series of inflammatory responses to remove pathogens and protect lung tissue. Pulmonary immune cells, such as dendritic cells, natural killer cells, macrophages, and neutrophils, maintain lung-tissue homeostasis (18). However, abnormal immune cell function can lead to continuous or excessive inflammatory reactions and subsequent lung tissue damage. Macrophages and neutrophils are additional immune cells that contribute to the pathogenesis of ALI/ARDS (18). Although macrophages play a key role in lung defense, their excessive activation aggravates lung damage. Various activation and death modes of macrophages, including polarization, apoptosis, autophagy, and pyroptosis et al., have different impacts on ALI/ARDS. Among them, pyroptosis is a newly discovered form of programmed cell death, depending on caspase-triggered cleavage of gasdermin proteins following recognition of the ligand in the cytoplasm (19). In ALI/ARDS caused by various factors, pulmonary damage is always accompanied by macrophage pyroptosis (20–22), suggesting that macrophage pyroptosis plays an essential role in ALI/ARDS.

At present, supportive treatment is the main therapeutic method for ALI/ARDS mainly including lung protective mechanical ventilation and fluid management therapy, supplemented by glucocorticoids, surfactants, and extracorporeal membrane oxygenation (23). Although supportive treatment can improve patients’ symptoms to some extent, it cannot significantly improve prognosis, instead the mortality remains high, and even patients in recovery may face long-term cognitive impairment and impaired quality of life (24). In addition, long-term or excessive mechanical ventilation may also lead to ventilator-induced lung injury(VILI)and even pulmonary fibrosis (25, 26). Therefore, it is of great significance to figure out the pathogenesis so that we can find much safer and more efficient treatment measures for ALI/ARDS.

## 3 Pyroptosis

### 3.1 Pyroptosis and its pivotal effector gasdermin

Pyroptosis is defined as gasdermin-mediated programmed cell death and closely related to a variety of diseases (27). Pyroptosis was first identified by Zychlinsky et al. when they first discovered suicide events in macrophages infected with the Gram-negative pathogen Shigella flexneri in 1992 (28). In the initial study, it was found that there were some similar characteristics between apoptosis and such a cell death way, such as caspase independence, DNA damage and nuclear condensation et al., so it had been called apoptosis for a long time. The subsequent studies showed it was different from apoptosis, especially in the orderliness of DNA fragments and the integrity of cell nucleus (29). In 2001, D’souza et al. first proposed the term of pyroptosis to describe this kind of programmed proinflammatory cell death, thereby distinguishing pyroptosis from apoptosis (30).

The human gasdermin protein family consists of six proteins: gasdermin A–E and deafness autosomal recessive type 59 (DFNB59, also called Pejvakin). Except for DFNB59, all gasdermin proteins contain two conserved domains including the N-terminal pore-forming domain (PFD) and the C-terminal repressor domain (RD) connected by a peptide linker (31). Normally, the PFD and RD of gasdermin proteins interact to maintain oligomerization so that the cytotoxicity of PFD can be inhibited by RD (32, 33). After the host is stimulated, the activation of upstream molecules such as caspase-1 and caspase-11/4/5 will trigger the cleavage of gasdermin, separating the PFD from the RD (34). Then the PFD oligomer binds to cell membranes, contributing to damage of cell membranes and formation of perforation, which further leads to cell swelling and release of inflammatory factors such as IL-1β and IL-18 (32, 35) and meanwhile perturbs regulation of ions and water (36). The formation of perforation, cell cleavage and release of proinflammatory cytokine are significant characteristics of pyroptosis (37).

**Figure 2:**
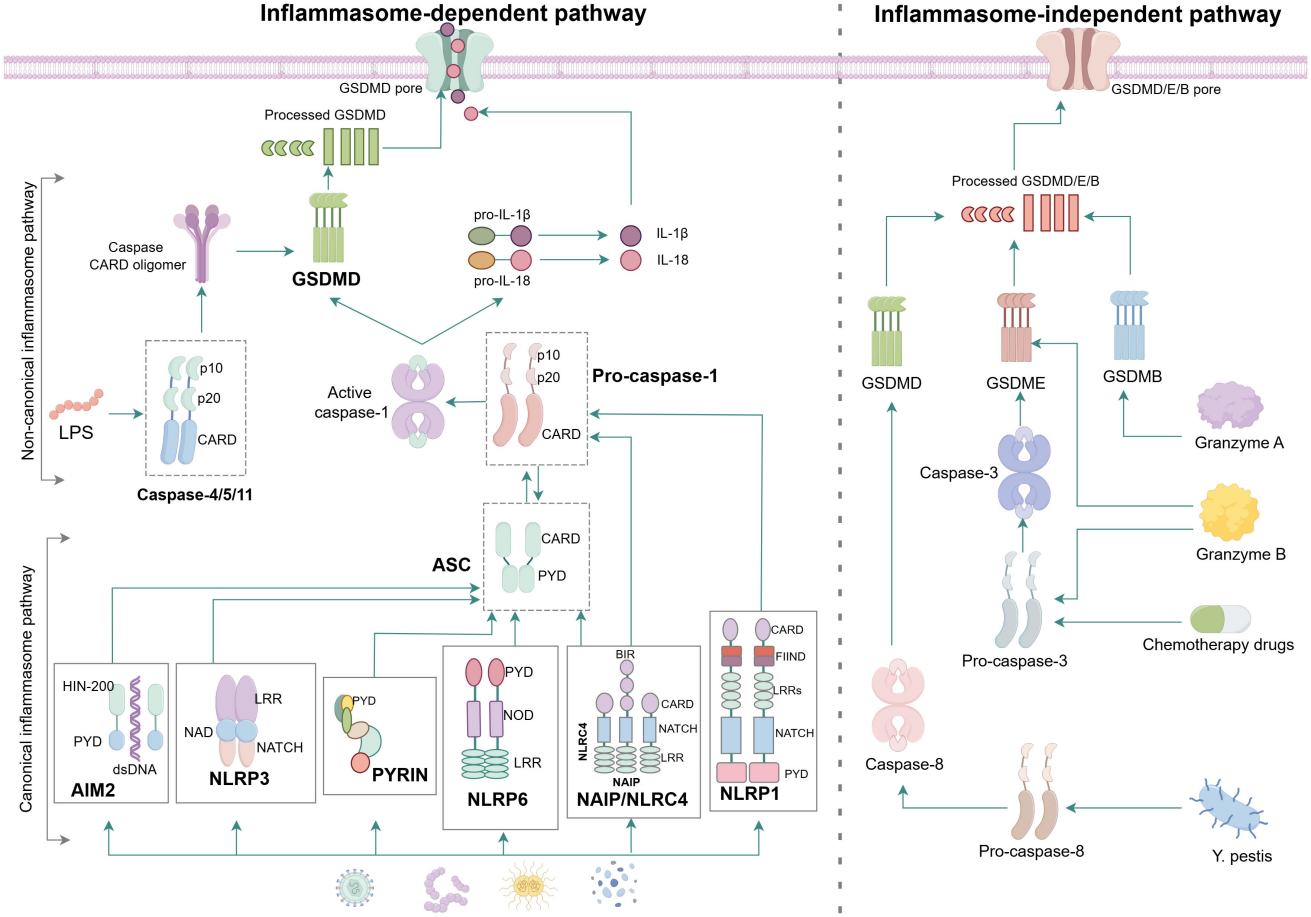
Molecular mechanism of pyroptosis. In the canonical inflammasome pathway, the majority of inflammasome complex is assembled from intracellular sensor protein, adapter proteins ASC and effector protein caspase. When PAMPs or DAMPs such as toxins, bacteria, viruses and dsDNA intrude, the NLRP1 and NAIP/NLRC4 can directly convert pro-caspase-1 into active caspase-1 because both of them have the CARD to connect with pro-caspase-1, while the AIM2, NLRP3, PYRIN and NLRP6 have to bind with ASC firstly before activating the pro-caspase-1 due to the lack of CARD. Active caspase-1 cleaves the GSDMD to causing damage to cell membrane and formation of perforations meanwhile cleaves pro-IL-1β and pro-IL-18 and results in the maturation of IL-1β and IL-18 that are subsequently released from the N-GSDMD pores. In the non-canonical inflammasome pathway, LPS directly binds to pro-caspase-4/5/11, resulting in activation of caspase-4/5/11, which cleaves GSDMD to trigger pyroptosis. In the granzyme-A/B-dependent pyroptosis pathway, GzmA and GzmB from NK cells and CD8 + T cells enter cancer cells via perforin and recognise GSDMB and GSDME, respectively, to induce pyroptosis. In addition, chemotherapeutic drugs and Y. pestis trigger pyroptosis through the caspase-3/GSDME or caspase-8/GSDMD cascades.

At present, there are three identified pathways of pyroptosis: canonical inflammasome pathway, non-canonical inflammasome pathway and inflammasome-independent pathway (**Figure 2**).

### 3.2 Canonical inflammasome pathway

The canonical inflammasome pathway is mediated by assembly of inflammasomes. As a large cytoplasmic multiprotein complex, the inflammasome is assembled in response to pathogens, damage-associated stimulation, and other danger signals that disrupt cell homeostasis (38, 39). It is capable of regulating the activation of caspase (40). The majority of inflammasome complexes consist of sensor protein (also called pattern recognition receptor, PRR), adopter protein(an apoptosis-associated speck-like protein [ASC] containing caspase activation and recruitment domain [CARD]) and effector caspase (pro-caspase-1) (41). ASC is a bipartite molecule containing a pyrin domain(PYD) and a caspase activation and recruitment domain (CARD), enabling itself to bridge the sensor and effector pro-caspase-1 (42, 43). The structure of caspase also contains a CARD, allowing ASC to interact with it through CARD-CARD coaction to recruit pro-caspase-1 for inflammasome assembly.

Several inflammasomes have been identified up to now including NLRP1, NLRP3, NAIP/NLRC4, AIM2, PYRIN and CARD8 et al. It is fundamental and essential for assembly of inflammasome complex to produce spirochetes through CARD-CARD or PYD-PYD interactions, which will mediate oligomerization of homology and heterogeneity (44, 45). As the first identified key component of inflammasome, the human NLRP1 contains an N-terminal PYD and a C-terminal CARD (46). NLRP3 contains three conserved domains including the nucleotide binding and oligomerization (NACHT) domain in the middle, the leucine-rich repeat domain (LRR) in the C terminus, and the pyrin domain (PYD) in the N terminus (47). When activated and assembled, NACHT domain acts as a support structure for oligomerization of NLRP3 to recruit ASC and pro-caspase-1 to form multiprotein inflammasome complex (48, 49). NAIPs are cytoplasmic receptors for various bacterial protein ligands, leading to recruitment of the adaptor protein NLRC4 to form the NAIP-NLRC4 inflammasome (50, 51). NLRC4 containing CARD can serve as an adaptor in the downstream of NAIP to recruit CASP1, and NAIP, together with NLRC4 and CASP1, is sufficient to initiate pyroptosis although it is essential to recruit ASC adaptor to the complex for processing of IL-1 and IL-18 (41). As one of the members of PYHIN protein family, AIM2 contains a PYRIN domain(PYD) and a HIN-200 DNA-binding domain. AIM2 differs from other inflammasomes components due to the lack of NBD and LRR, but it can still coordinate the oligomerization of large inflammasome complexes by the way that AIM2 interacts with ASC via PYD (52), and the downstream pathway, including the processing of cytokines and pyroptosis, is similar to that of proteins containing NBD-LRR domains. The structure of PYRIN includes the N-terminal PYD, b30.2/SPRY domain, and central B box and coiled-coil domain, whose mode of action is similar to NLRP3. At present, NLRP12, NLRP6 and NLRP9b are also considered to be involved in the inflammasome complex, and NLRP6 is similar in structure and assembly to NLRP3. Overall, the assembly of NLRP3, NLRP6, AIM2 and PYRIN is strictly dependent on the adaptor protein ASC while NLRP1, NLRC4 and CARD8 can induce inflammasome assembly and the subsequent pyroptosis in an ASC-independent way because of the existence of CARD. However, participation of ASC is essential for NLRC4 to mediate the processing and release of cytokines.

### 3.3 Non-canonical inflammasome pathway

In the non-canonical inflammasome pathway, caspase-11/4/5(caspase-11 is the mouse homologue of human caspase-4/5)can be activated by the way that the N-terminal CARD binds in direct to intracellular lipopolysaccharide(LPS) (53), and activated caspase-11/4/5 can also cleave gasdermin D(GSDMD) to N-GSDMD, oligomerizing it and transferring it to the cell membrane, ultimately forming membrane perforation (54). LPS is a major component of the outer membrane of Gram-negative bacteria and consists of three parts including the most conserved lipid moiety, a core oligosaccharide chain and a variable polysaccharide chain known as O-antigen (55). Although caspase-11/4/5 contains a CARD like caspase-1, their binding to LPS requires some specific charged residues. The predicted isoelectric point of CARD of caspase-11/4/5 is alkaline(>8) while that of caspase-1’s is about 6, which explains the binding between the CARD of caspase-11/4/5 and the acidic phosphate of the lipid A skeleton in LPS (53, 56). However, pro-IL-1β and pro-IL-18 can not be cut by caspase-11/4/5. In some cells, such as monocyte, GSDMD cleaved by caspase-11/4/5 generates efflux of K^+^, inducing assembly of NLRP3 inflammasome and then mediating maturement and secretion of IL-1β and IL-18 through NLRP3/caspase-1 pathway (27, 57, 58).

### 3.4 Inflammasome-independent pathway

In addition to the above pathways, some studies have shown that with treatment of chemotherapeutic drugs the gasdermin-E(GSDME) when highly expressed can be characteristically cleaved and activated by caspase-3 to form N-GSDME termini, leading to pyroptosis of tumor cells (59, 60). Furthermore, PD-L1 transfers apoptosis mediated by TNF into pyroptosis in breast cancer cells and the main mechanism is that the p-STAT3 promotes nuclear translocation of PD-L1 as well as transcription of gasdermin-C(GSDMC) and then under the stimulation of TNF-α, caspase-8 specifically cleaves GSDMC to generate N-GSDMC, forming holes in cell membrane to induce pyroptosis (61).

Granzyme, a serine protease, is released from cytosolic granules of cytotoxic lymphocytes (CTLs) and natural killer cells (NK). Granzyme A from CTLs can cleave and activate gasdermin-B(GSDMB) and then release its pore-forming activity, triggering pyroptosis in target cells (62). Besides, CAR-T cell is able to rapidly activate caspase-3 in target cells by releasing granzyme B(GZMB), subsequently triggering caspase-3/GSDME-mediated pyroptosis (63).

Cell pyroptosis can trigger both immune protection, pathological inflammation and tissue damage (64). Overall, pyroptosis depends on the members of the gasdermin protein family to facilitate the formation of membrane pores, resulting in the release of pro-inflammatory mediators and cellular contents, which ultimately contributes to cell disruption. Under normal circumstances, these mediators play a vital role in activating and regulating the immune response, helping to control or eliminate invading pathogens. In ALI/ARDS, excessive release of cytokines and infiltration of immune cells can trigger a cytokine storm, leading to severe damage to lung tissue (5). In the case of ARDS, IL-1β has emerged as a potent pro-inflammatory factor found in the lungs of affected patients (65). Additionally, the cytokines released in pyroptosis promote the polarization of macrophages into M1 phenotype, aggravating the inflammatory response in the lungs (66).

## 4 Macrophage

### 4.1 The origin of macrophage

Macrophages are important components of the innate immune system and the activation of macrophage has been proven to be essential for immune defense, inflammatory response, tissue remodeling and homeostasis et al (67). Macrophages were first identified and named by immunologist Ellie Metchnikoff according to the characteristics of swallowing and killing bacteria in 1882 (13). At the very beginning, it was thought that macrophages originate from bone marrow-derived monocytes that are recruited to the tissue and differentiate into macrophages within tissue (68). Later research shows that the contribution of blood monocytes to the macrophage population homeostasis appears to be limited to a few specific tissues while a great number of tissue macrophages such as brain microglia, liver Kuffer cells, heart macrophages and alveolar macrophages originate from yolk sac or original macrophages existing in fetal livers and are maintained independently of blood monocytes (69, 70). Macrophages have the functions of phagocytosis, antigen presentation and immune defense and regulation (71, 72). In different environments and under various stimulations, macrophages will develop processes such as autophagy (73), polarization (67) and pyroptosis (74) to maintain tissue homeostasis. Based on their function and activation, macrophages are divided into two subtypes: classically activated M1 macrophages which produce mainly proinflammatory factors as part of the immune defense response and alternative activated M2 macrophages which mainly secrete anti-inflammatory factors to promote tissue repair (67, 75).

### 4.2 Pulmonary macrophage

**Figure 3:**
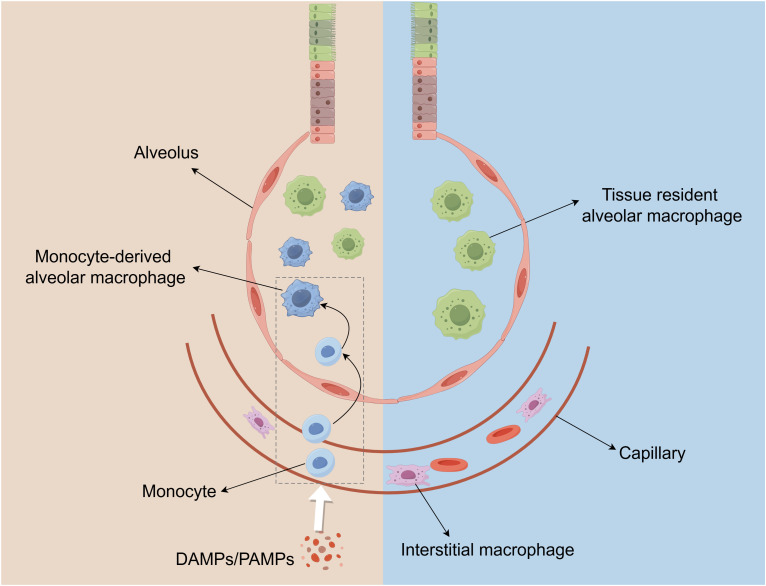
The distribution of macrophage populations in the lung. Macrophages in the lungs are composed of Alveolar macrophages (AMs) and interstitial macrophages (IMs). AMs are located in the alveoli, and IMs reside in the parenchyma between the microvascular endothelium and alveolar epithelium. When there is pulmonary inflammation and injury, circulating monocytes in capillaries are subsequently recruited to the lungs and converted into AM-like cells.

There are two subtypes of pulmonary macrophages including alveolar macrophages(AM) and pulmonary interstitial macrophages(IM) (76), and each type of macrophage contains tissue-resident and recruited macrophages, which are critical participants in innate immune and maintaining pulmonary homeostasis (**Figure 3**) (77). Acute inflammation and severe infection typically lead to loss of tissue-resident macrophages and recruitment of monocytes-derived macrophages, which become part of the pulmonary macrophage repertoire (77). The current study revealed that tissue-resident interstitial macrophages and monocyte-derived interstitial macrophages exhibit distinct profiles in omics analysis, but no significant differences were observed in their functional characteristics and properties (78).

AM is an important cell line for catabolism of surface active material produced by alveolar type II epithelial cells and essential effector cells resisting external stimuli in lung, playing a critical role in the pathogenesis of pulmonary inflammation (79, 80). Generally, AM is able to continuously capture, swallow, conceal and neutralize vast inhaled pathogens and particles, which will not induce excessive inflammation triggered by influx of neutrophils (81). But when such a swallowing function is surpassed, AM will induce inflammation by producing chemokines and cytokines (such as type I IFNs, TNF-α, and IL-1β) that can recruit and activate neutrophils, monocytes and DCs (81). Tissue-resident alveolar macrophages(TR-AMs) and monocyte-derived alveolar macrophages(Mo-AMs) exhibit distinct characteristics during both homeostasis and disease states. TR-AMs play a crucial role in the clearance of dead alveolar cells and excess alveolar surfactants (82). In the absence of inflammation or tissue damage, TR-AMs maintain their cellular population through local proliferation, thereby minimizing the contribution of Mo-AMs (83). Upon the onset of inflammation, TR-AMs are rapidly activated, releasing a variety of cytokines and chemokines that modulate the inflammatory response and influence tissue integrity (84). Concurrently, the inflammatory process triggers the recruitment of monocytes to the lungs, where they differentiate into macrophages within the alveolar space, potentially exacerbating tissue damage through the release of pro-inflammatory mediators (85). Additionally, the time and cause of pyroptosis may vary among different macrophage populations. AMs emerged as the earliest cell type to initiate pyroptosis and act as pivotal regulators of cell communication (86). AMs usually die early in ALI/ARDS, and some pathogens can directly induce pyroptosis. *In vitro* experiments, the level of macrophage pyroptosis in RAW264.7 cells increased significantly within 24 hours after stimulation with LPS (87). The NETs and some cytokines can induce pyroptosis of monocyte-derived macrophages in the mid-stage (66, 88). In general, apoptosis is an important process in tissue homeostasis, which eliminates excessive cells in multicellular organisms, while pyroptosis usually leads to severe inflammatory response and tissue damage, aggravating disease progression. Although macrophage pyroptosis represents a distinct form of cell death compared to macrophage apoptosis, these two processes are not entirely independent. It has been identified that GSDME and GSDMD can induce the transition from apoptosis to pyroptosis. Caspase-3 and caspase-1, as the main performers of apoptosis and pyroptosis respectively, were also found to have crosstalk (89). However, there is uncertainty existing about whether apoptosis induced by pyroptosis protects cells from the pyroptosis-induced inflammatory response or further accelerates the inflammatory response of macrophages.

## 5 Macrophage pyroptosis and ALI/ARDS

### 5.1 Macrophage pyroptosis aggravates ALI/ARDS

Pyroptosis is a double-edged sword. For one thing, it enhances adaptive immune response by releasing pro-inflammatory medium to gather the nearby immune cells around the pathogen-infected place and then destroy the replication niches (90). For another thing, excessive pyroptosis will aggravate multiple organ damage, circulatory failure and even death (91). Most of the time, macrophage pyroptosis significantly aggravates the progression of ALI/ARDS primarily through exacerbating pulmonary inflammation and tissue damage. Studies revealed that the mean caspase-1 levels, which are associated with pyroptosis, were significantly elevated in patients with ARDS compared to healthy individuals. It is noteworthy that the mean caspase-1 levels in nonsurviving patients were also significantly higher than those in survivors. Concurrently, the levels of inflammatory cytokines, including IL-1β, IL-18, and TNF-α, were significantly elevated and the extent of pulmonary injury was more severe in patients with ARDS compared to healthy controls (92, 93). In sepsis-induced acute lung injury, the incidence of macrophage pyroptosis were increased, followed by overexpression of pro-inflammatory mediators such as iNOS, IL-1β and TNF-α, and observable pulmonary tissue lesions (94).

Macrophages are capable of recognizing PAMPs and DAMPs, thereby initiating a cascade of responses during pyroptosis. Additionally, the impact of macrophage pyroptosis on ALI/ARDS is also modulated by protein post-translational modifications (95), mitochondrial function (96), exosome activity (97), neutrophil extracellular traps (98) and other various signaling pathways (99–101). The above factors can exert a positive or negative influences on the regulation of macrophage pyroptosis. Generally, modulating these factors to inhibit macrophage pyroptosis holds significant potential for mitigating ALI/ARDS.

### 5.2 Inhibition of macrophage pyroptosis alleviates ALI/ARDS

#### 5.2.1 Inhibiting macrophage pyroptosis by regulating mitochondrial function reduces ALI/ARDS

Mitochondria are not only the major sites of cellular energy production through oxidative phosphorylation but also the participants in various cellular processes (96, 102). Studies have confirmed that mitochondria are involved in regulation of macrophage pyroptosis in ALI/ARDS.

Mitophagy plays a crucial role in mitochondrial quality control and cell survival by selectively eliminating excess or damaged mitochondria through the autophagic process (103). The present study indicates that mitophagy contributes to the mitigation of macrophage pyroptosis in ALI/ARDS. Defects in mitophagy result in the accumulation of mitochondrial reactive oxygen species (mtROS), leading to the overactivation of the NLRP3 inflammasome, and subsequently triggering Caspase-1-dependent pyroptosis (104). Dong et al. verified that the miR-138-5p promoter demethylation attenuates the pyroptosis of AMs in sepsis-associated ALI while increased mitophagy reduces cytoplasmic mtDNA levels, suppressing miR-138-5p promoter methylation, which suggested that enhanced mitophagy can reduce AM pyroptosis and alleviate sepsis-associated ALI (105).

Conversely, mitochondrial damage led to an increase of macrophage pyroptosis. Mitochondrial damage-associated molecular patterns (MTDs) are a type of damage-associated molecular patterns (DAMPs) that are released from mitochondrial rupture (106). MTDs have been reported to induce NLRP3 inflammasome activation, resulting in severe inflammatory response in alveolar macrophages (107). In sepsis-induced ALI, Z-DNA-binding protein 1 (ZBP1) deficiency in macrophages mitigates mitochondrial damage, consequently reducing macrophage pyroptosis mediated by NLRP3 inflammasome activation (108).

Additionally, the study conducted by Han et al. identified that the 18-kDa translocator protein (TSPO), a mitochondrial outer membrane protein, is a crucial mediator regulating NLRP3 inflammasome activation in macrophages during ALI/ARDS (109). They found that the expression of TSPO was rapidly upregulated in response to inflammatory stimulation and its deficiency resulted in enhanced activation of the NLRP3 inflammasome pathway in LPS-injured lung tissue (109).

#### 5.2.2 Inhibition of macrophage pyroptosis by regulating exosome reduces ALI/ARDS

Exosome is the nanoscale membrane-bound extracellular vesicle that plays crucial roles in intercellular communications by carrying bioactive molecules, such as proteins, RNAs, microRNAs (miRNAs) and DNAs, from one cell to the others (97). Crosstalk between exosome and inflammasome activation has been verified in many studies. As the upstream of inflammasome, exosome can either promote or suppress activation of inflammasome, subsequently affecting macrophage pyroptosis and ALI/ARDS. This discrepancy of exosome effects is likely affected by the type of the cells producing exosomes and interventions or conditions that induce cells to release the exosomes (97, 110).

Polymorphonuclear neutrophils (PMN) play an important role in ALI/ARDS, and exosomes derived from it are a new subcellular entity which is basic links between inflammation and tissue damage driven by PMN (111). The study shows that TNF-α-stimulated exosomes(TNF-Exo) derived from PMN is able to transfer the miR-30d-5p of miRNA family into macrophages and then activate the NF-κB signaling to up-regulate the expression of NLRP3 inflammation, which triggers pyroptosis in macrophages and therefore promotes sepsis-associated ALI (94). Another kind of exosome called Tenascin-C(TNC) induces macrophage pyroptosis by DNA damage response. The specific mechanism is that the DNA damage response could be induced by excessive TNC-produced ROS and activation of p38/ERK/NF-κB signaling to trigger macrophage pyroptosis to make ALI/ARDS severe (112). However, exosomes derived from mesenchymal stem cells(MSCs-Exo) can inhibit AM pyroptosis by down-regulation activated caspase-1, thereby alleviating LPS-induced ALI (113). Bone marrow mesenchymal stem cells (BMSCS) -derived exosomes can deliver miR-125b-5p and reduce macrophage pyroptosis by regulating STAT3 expression, subsequently improving sepsis-induced acute lung injury (114).

In conclusion, although exosomes from different cells have different impacts on macrophage pyroptosis, regulating exosomes can inhibit macrophage pyroptosis and reduce ALI/ARDS.

#### 5.2.3 Inhibition of macrophage pyroptosis by regulating neutrophil extracellular traps reduces ALI/ARDS

Neutrophils are short-lived granulocytes, serving as the primary line of defense against pathogens (115). Activated neutrophils release neutrophil extracellular traps (NETs) in response to various stimuli, which was identified as part of innate immune response, and this response can either be beneficial or pathological (98, 116).

In ALI/ARDS, NETs aggravate lung injury mainly by promoting macrophage pyroptosis (90, 93). The study proved that neutrophils undergo NETosis to produce a large amount of NETs. These NETs are engulfed by alveolar macrophages, leading to AIM2 inflammasome activation and caspase-1-dependent pyroptosis in LPS-priming alveolar macrophages. This, in turn, results in the release of a large amount of cytokines and more neutrophil infiltration, leading to ARDS through inflammatory storm development due to a vicious cycle (93). A study conducted by Liu et al. found that pretreatment of alpha-linolenic acid(ALA) might alleviate NETs-induced alveolar macrophage pyroptosis by mediating Pyrin inflammasome activation, which alleviated ALI/ARDS (117). They utilized Mefv (Pyrin)-/- mice to demonstrate that NETs induce macrophage pyroptosis depending on Pyrin inflammasome. In addition, The research found that IgG IC-induced formation of NETs stimulates pulmonary macrophage pyroptosis in ALI/ARDS, which could be inhibited by verbenalin (118).

#### 5.2.4 Inhibiting macrophage pyroptosis by regulating protein post-translational modifications reduces ALI/ARDS

Post-translational modifications (PTMs) refer to a series of covalent modifications of proteins following the translation of RNA, constituting a critical phase in protein biosynthesis (119). Throughout the life cycle, PTMs contribute to increasing complexity of the proteome, modulating subcellular localization of associated proteins, facilitating or inhibiting protein-protein interactions, and activating or inactivating target proteins (120). Recent reports have identified multiple PTMs in pyroptosis process. For example, (de)ubiquitylation, (de)phosphorylation, (de)SUMOylation, (de)acetylation, alkylation and citrullination are involved in the assembly and activation of inflammasomes (NLRP3, NLRC4, AIM2) (121–123), while ubiquitination, phosphorylation and ADP- ribosylation participate in caspase activation, which is essential for the cleavage of GSDMD (124). Additionally, several PTMs can directly modulate the cleavage of GSDMD, such as ubiquitylation, phosphorylation, palmitoylation and succinylation, while ubiquitylation exerts a negative regulatory effect on the release of mature IL-1β and IL-18 through the pores formed by GSDMD-N (125, 126).

The majority of PTMs are reversible. For instance, deubiquitinating enzymes (DUBs) can reverse ubiquitination by hydrolyzing the peptide or isopeptide bonds that link ubiquitin molecules to each other or to substrate proteins. Ubiquitination typically suppresses inflammasome activity, thereby inhibiting pyroptosis, while deubiquitination has the opposite effect (127, 128). SUMO-conjugating enzyme (UBC9) interacts with NLRP3 and promotes SUMO1 to catalyse SUMOylation at Lys204 in NLRP3, subsequently promoting NLRP3 activation and macrophage pyroptosis. Conversely, SUMO-specific protease 3 (SENP3) catalyses deSUMOylation of NLRP3, reducing ASC recruitment, ultimately suppressing NLRP3 inflammasome activation, as well as IL-1β cleavage and secretion (129). However, there are also some PTMs that are irreversible, such as citrullination (130). The current studies suggest that protein citrullination can promote macrophage pyroptosis through regulating activation of inflammasomes and caspase proteins, thereby aggravating ALI/ARDS (131, 132).

It has been demonstrated that Heat Shock Factor 1 (HSF1) mitigates sepsis-induced lung injury through the promotion of NLRP3 ubiquitination in macrophages (122). In human THP-1 cells and mouse bone marrow-derived macrophages lacking the DUB USP50, reduced activation of pyroptosis was observed, leading to decreased levels of IL-1β and IL-18. Further investigations revealed that USP50 interacts with ASC proteins and deubiquitinates K63-linked ASC polyubiquitination (133). Immunostaining of human macrophages indicates that the bacterial effector protein NLeA inhibits inflammasome activity through its binding to ubiquitinated NLRP3, thereby disrupting the deubiquitination process. This interaction prevents inflammasome assembly, caspase-1 activation, and subsequent IL-1β secretion (134). The peptidylarginine deiminase 2(PAD2) is a calcium-dependent enzyme that promotes the conversion of arginine to citrulline. It has been demonstrated that PAD2 knockout can reduce the mortality of Pseudomonas aeruginosa induced pneumonia mice by reducing the caspase-1-dependent inflammasome activation in macrophages (135). In sepsis-associated acute lung injury, PAD2 deficiency decreased caspase-11-dependent macrophage pyroptosis, increasing survival and organ functions following the onset of sepsis (132). Furthermore, the study found that inhibition of PAD2 leads to a reduction of ASC citrullination, suggesting that PAD2 may influence inflammasome assembly through the mediation of ASC citrullination, ultimately impacting macrophage pyroptosis (136). NLRC4 is a cytosolic member of the NOD-like receptor family that is expressed in innate immune cells. The phosphorylation of residue Ser 533 in NLRC4 had been identified to activate NLRC4 inflammasome activity and induce conformational changes that are essential for host immune responses (137).

The effect of PTMs on caspase protein and gasdermin protein is not as extensive as that on inflammasome. There is a study demonstrating that the E3 ubiquitin ligase, synoviolin (SYVN1), mediates the K27-linked polyubiquitination of GSDMD at K203 and K204, promoting GSDMD-induced pyroptosis (138). In addition, upon activation by inflammasome, palmitoyl transferases ZDHHC5/9 can induce S-palmitoylation at Cys191 (human)/Cys192 (mouse) on GSDMD, generating palmitoylated GSDMD-N which effectively triggers pyroptosis (139).

#### 5.2.5 Inhibition of macrophage pyroptosis by regulating nuclear factor-erythroid 2 related factor 2 pathway reduces ALI/ARDS

Nuclear factor-erythroid 2 related factor 2(Nrf2)is a ubiquitous master transcription factor that upregulates antioxidant response elements (AREs)-mediated expression of antioxidant enzymes and cytoprotective proteins (140). Activation of Nrf2 has been shown to be protective against lung injury (140), and increasing evidence also demonstrates the crosstalk between the Nrf2 and NLRP3 inflammasome axis at different levels (141). Liu et al. found that Nrf2 knockdown can substantially increase the mRNA level of NLRP3 (142). Further studies showed that upregulating of AMPK phosphorylation can promote expression of Nrf2 followed by inhibition of NLRP3 transcription, thereby suppressing pyroptosis in alveolar macrophages and ultimately alleviating ALI/ARDS (142). In the models of LPS-induced sepsis, melatonin inhibits NLRP3-GSDMD pathway via activating Nrf2/HO-1 signaling axis to reduce ALI/ARDS *in vivo* and *in vitro* (100). In addition, Nrf2 is able to inhibit activation of NLRP3 inflammasome by reducing intracellular ROS levels and avoiding inflammation (143). A study showed that chicoric acid alleviated NLRP3-mediated macrophage pyroptosis in the ALI model. This effect was achieved through ROS-induced mitochondrial damage by activating the Akt/Nrf2 pathway via PDPK1 ubiquitination (144). In conclusion, it is of great significance in reducing ALI/ARDS to inhibit activation of NLRP3 by up-regulating Nrf2 level.

#### 5.2.6 Inhibition of macrophage pyroptosis by regulating stimulator of interferon genes pathway reduces ALI/ARDS

Stimulator of interferon genes(STING)is a signaling molecule associated with the endoplasmic reticulum (ER) and is essential for controlling the transcription of numerous host defence genes, including type I interferons (IFNs) and pro-inflammatory cytokines, following the recognition of aberrant DNA species or cyclic dinucleotides (CDNs) in the cytosol of the cell (145, 146). STING signaling has now been shown to be essential for protecting the cell against a variety of pathogens (147). At present, growing studies have shown that STING involved in regulation of macrophage pyroptosis mainly by affecting NLRP3 inflammasome activation in ALI/ARDS. Peng et al. found that up-gratulation of STING signaling can promote NLRP3-mediated pyroptosis in macrophages and knockout of cGAS/STING could ameliorate NLRP3 activation and macrophage pyroptosis, ultimately improving SAP-ALI in mouse model (148). In LPS-induced ALI, LPS could activate STING in a cytosolic DNA-dependent manner and upregulate the expression of STING in a c-Myc-dependent manner, which cooperatively promote NLRP3-mediated macrophage pyroptosis following contributing to acute pulmonary damage (149). Furthermore, another study suggested that histone deacetylase 3 (HDAC3) activates cGAS/STING pathway through its histone deacetylation function, playing an essential role in mediating macrophage pyroptosis and ALI (101). The specific mechanism is that HDAC3 and H3K9Ac are recruited by LPS to the miR-4767 gene promoter, which repressed expression of miR-4767 to promote the expression of cGAS (101).

#### 5.2.7 Inhibition of macrophage pyroptosis by regulating nuclear factor kappa-B pathway reduces ALI/ARDS

Nuclear factor kappa-B(NF-κB)is a Rel family transcription factor consisting of five members in mammalian cells, namely RelA (p65), RelB, Rel (c-Rel), NF-κB1 (p50/p105) and NF-κB2 (p52/p100) (150), which controls both innate and adaptive immune responses as well as the development and maintenance of the cells and tissues that comprise the immune system (151). NF-κB is widely recognized as a key regulator of inflammation due to its pivotal role in governing diverse aspects of the inflammatory response, including evolution and resolution (152). NF-κB signaling is also involved in the progression of ALI/ARDS (153, 154), and NLRP3 inflammasome activation requires involvement of NF-κB (122). Triggered by PAMPs, TNF, and IL-1β, the mRNA expression of NLRP3 and pro-IL-1β is upregulated by activating NF-κB. In septic ALI, heat shock factor 1 (HSF1) inhibited the NF-κB signaling pathway by upregulating tumor necrosis factor receptor-associated factors 3(TRAF3)expression, thereby inhibiting the production of NLRP3 at the transcriptional level and ultimately inhibiting the activation of the NLRP3 inflammasome, which reduces macrophage pyroptosis and subsequently alleviates pulmonary damage (122). Sun et al. discovered that angiopoietin-like 4 (ANGPTL4) silencing could disrupt the activation of the NF-κB pathway to repress the pyroptosis and polarization of M1 macrophages, whereby suppressing the CLP-induced sepsis-related ALI (155). Other studies demonstrated that macrophage pyroptosis mediated by NLRP3/GSDMD signaling can be inhibited by suppressing the NF-κB activation (99, 156), suggesting that inhibiting pyroptosis of macrophages by targeting NF-κB pathway can reduce acute lung injury. In addition, S100A9 gene deficiency inhibits pyroptosis of macrophages through TLR4/MyD88/NF-κB pathway, alleviating LPS-induced acute lung injury (157), which suggests a potential therapeutic strategy for the treatment of ALI/ARDS.

#### 5.2.8 Other pathways that can reduce ALI/ARDS via inhibiting macrophage pyroptosis

Apart from the above regulatory modes, there are many other signaling pathways that can affect macrophage pyroptosis in ALI/ARDS. A body of evidence confirmed that p38 mitogen-activated protein kinase (MAPK) signaling pathway participates in the progression of ALI/ARDS (158, 159). Li et al. demonstrated that macrophage cell death could be skewed from proinflammatory pyroptosis towards non−inflammatory apoptosis through blockage of p38 MAPK signaling pathway, which may contribute to amelioration of acute lung injury and excessive inflammation in mice of ALI induced by LPS (160).

Tumor necrosis factor receptor-associated factor 3 (TRAF3) is the member of TRAF family, playing a crucial role in regulating both immune and inflammatory response. The study showed the TRAF3/ULK1/NLRP3 axis promoted the development of ALI in mice by inducing alveolar macrophage pyroptosis. The vitro cell experiments verified that TRAF3 can downregulate ULK1 through ubiquitination and activate the NLRP3 inflammasome to induce alveolar macrophage pyroptosis (161).

Basic helix-loop-helix family member e40 (Bhlhe40), belonging to the subfamily of transcription factors, is considered as an important regulator of inflammation and immunity (162). A study showed the expression of Bhlhe40 significantly increased in AMs treated with LPS. Meanwhile, Bhlhe40−/− mice exhibited decreased macrophages pyroptosis and inflammation by inhibiting signaling pathway mediated by caspase-1 and caspase-11, and they were resistant to LPS-induced ALI (163).

## 6 Advances in drugs application to improve ALI/ARDS by interfering macrophage pyroptosis

### 6.1 Natural small-molecule compounds

Natural products derived from plants play an important role in the treatment of ALI/ARDS. Many studies show that natural active ingredients from Chinese herbs can regulate pyroptosis in macrophages, alleviating ALI/ARDS.

Alpha-linolenic acid (ALA) is a plant-based omega-3 fatty acid. Studies show that ALA reduces ALI/ARDS by inhibiting the activation of macrophage pyroptosis driven by Pyrin inflammasome (117). Emodin, a natural active ingredient extracted from the Chinese herb Radix et Rhizoma Rhei, alleviates LPS-induced ALI by inhibiting the NLRP3 inflammasome-dependent pyroptosis signaling pathway of macrophages *in vitro* and *in vivo* (164). Arctiin (ARC) is a lignan glycoside isolated from Fructus arctii that exerts strong anti-inflammatory and antioxidant effects (165, 166). Studies demonstrated that a nanoparticle (NP)-based delivery system, ARC@DPBNP, could be applied to reduce LPS-induced acute lung injury through attenuating pyroptosis in alveolar macrophages (167). Sinensetin (SIN) is a polymethoxylated flavonoid, which is proven to improve LPS-induced ALI by inhibiting Txnip/NLRP3/Caspase-1/GSDMD signaling-mediated macrophage pyroptosis. Dehydroandrographolide(Deh), as one of main components of Andrographis paniculata (Burm.f.) Wall, can weaken ROS production in mitochondria to suppress NLRP3-mediated pyroptosis in macrophages, thereby alleviating ALI/ARDS (168). Shao et al. found that Britannin extracted from Inula japonica Thunb. is an innate inhibitor that effectively targeted NLRP3, suppressing activation of NLRP3 inflammasome in an NF-κB-independent manner and inhibiting assembly of the NLRP3 inflammasome by directly binding to the NACHT domain of NLRP3 (169), which suggests that Britannin may be an effective drug inhibiting macrophages to reduce ALI/ARDS.

### 6.2 Synthetic small-molecular compounds

Buformin (BF), a number of the biguanide family, was originally used clinically as a hypoglycemic agent in the treatment of type 2 diabetes (170). Studies conducted by Liu et al. demonstrated that BF can reduce sepsis-induced lung injury by inhibiting NLRP3-mediated macrophages pyroptosis in an AMPK-dependent manner *in vivo* and *in vitro* (142). Sacubitril/valsartan (SV) is an angiotensin receptor-neprilysin inhibitor used for treating heart failure in the clinical settings (171). The study showed that SV treatment effectively alleviated sepsis-induced lung injury in caecal ligation and puncture (CLP) mice (172). Further study found that the SV could inhibit GSDMD-mediated macrophage pyroptosis through the caspase-1-dependent signaling pathway, contributing to the resolution of the inflammatory response and lung injury in sepsis (172). As an N-methyl-D-aspartic acid receptor (NMDAR) antagonist, memantine suppresses macrophage pyroptosis through inhibiting NLRP3 inflammasome (173). In cardiopulmonary bypass (CPB)–induced ALI, hydromorphone (Hyd) alleviated NLRP3 inflammasome-mediated pyroptosis via upregulating the Nrf2/HO-1 pathway, which may be achieved by AMs (174).

### 6.3 Other promising related compounds or interventions

Apart from medications, there are alternative interventions that can mitigate ALI/ARDS by suppressing macrophage pyroptosis. More and more studies have confirmed it is a promising strategy for ALI/ARDS to regulate exosomes. Liu et al. demonstrated that mesenchymal stem cells-derived exosomes(MSCs-Exo) can alleviate acute lung injury by inhibiting alveolar macrophage pyroptosis (113). The specific mechanism is MSCs-Exo inhibited J774A.1 cell pyroptosis by inhibiting the activation of caspase-1 (113). In addition, exosomes from bone marrow-derived mesenchymal stem cells, serving as carriers for delivering miR-125b-5p, can downregulate STAT3, thereby inhibiting macrophage pyroptosis and alleviating sepsis-associated ALI (114). The above studies suggest that the regulation of mesenchymal stem cell-derived exosomes can effectively inhibit macrophage pyroptosis and alleviate ALI/ARDS.

In LPS-induced ALI/ARDS, researchers found that NETs directly promoted alveolar macrophage pyroptosis through NET DNA-mediated activation of the AIM2 inflammasome, suggesting that NETs and the AIM2 sensor may be crucial therapeutic targets for the regulation of alveolar macrophage inflammasome-mediated immunopathology in ARDS (93). Melatonin (Mel, N-acetyl-5-methoxytryptamine) is a neurosecretory hormone, as well as a potential modulator of Nrf2 signaling based on its abilities to scavenge ROS and inflammatory cytokines (175). The study proved that melatonin significantly inhibits LPS-induced pyroptosis, attributed to its regulation of NLRP3-GSDMD pathway via activating Nrf2/HO-1 signaling axis (100). Irisin, a hormone−like myokine, can attenuate ALI by inhibiting the HSP90/NLRP3/caspase−1/GSDMD signaling pathway, reducing the pyroptosis of macrophages (176). The following section discusses the treatments and mechanisms related to macrophage pyroptosis in ALI/ARDS (**Table 1**).

**Table 1:** The treatment and mechanisms of macrophage pyroptosis in ALI/ARDS.

Animal/cell	Model	Treatment	Macrophage Pyroptosis	Mechanism	Reference
**Mouse**	LPS	TSPO-KO	−	TSPO↑, NLRP3↓	(102)
**Mouse**	*Klebsiella pneumoniae infection*	Anthocyanin	−	mtDNA↓, NLRP3↓	(177)
**Mouse**	MTDs	Autophagic agonist	−	Autophagosomes↑, NLRP3↓, ASC↓, pro-caspase-1↓	(105)
**Mouse**	CLP	TNF-Exo	+	NF-κB pathway↑, NLRP3↑, IL-1β↑, GSDMD-N↑	(110)
**Cell**	LPS	Tenascin-C	+	ROS↑, p38/ERK/NF-κB↑, dsDNA↑, Caspase-1↑, GSDMD↑, GSDME↑, AIM2↑	(111)
**Mouse** **Cell**	LPS LPS/Nig	MSCs-Exo	−	Caspase-1↓, IL-18↓, IL-1β↓	(94)
**Cell** **Mouse**	LPS/ATP LPS	BMSCs-Exo	−	STAT3↓, p-STAT3↓, NLRP3↓, GSDMD↓, caspase-1↓…	(112)
**Mouse**	LPS	Alpha-linolenic acid	−	NETs↓, caspase-1↓, GSDMD↓, ASC↓, IL-1β↓	(116)
**Mouse** **Cell**	LPS	Buformin	−	AMPK↓, mTOR↓, autophagy↑, Nrf2↑, NLRP3↓	(140)
**Mouse**	LPS	Melatonin	−	Nrf2/HO-1↑, NLRP3↓, GSDMD↓	(141)
**Mouse**	LPS	Chicoric acid	−	AKT/Nrf2↑, NLRP3↓	(100)
**Mouse**	SAP	cGAS-KO, STING1-KO	−	NLRP3↓	(146)
**Mouse**	CLP	HSF1-KO	+	NF-κB pathway↑, NLRP3↑, IL-1β↑IL-10↑	(153)
**Mouse** **Cell**	LPS	PAI-1 KO	+	PI3K/MAPK/AKT↑, NET↑, NLRP3↑, ASC↑, caspase-11↑, pro-caspase1↑, caspase1-p20↑, IL-1β↑	(159)
**Mouse**	LPS	Bhlhe40 KO	−	GSDMD↓, caspase-1↓, caspase-11↓	(161)
**Cell**	LPS	USP50 knockdown	−	ASC oligomerization↓,procase 1↓, IL-1β↓, IL-10↓	(133)
**Mouse**	CLP	PAD2 inhibition	−	ASC citrullination↓, NLRP3↓, caspase-1↓, caspase-11↓	(132)

The symbol ↑ indicates an increase and the ↓ indicates a decrease.

### 6.4 Challenges in alleviating ALI/ARDS through targeting macrophage pyroptosis

Undoubtedly, the therapeutic intervention of targeted macrophage pyroptosis is still challenging to some extent. Although the mechanism of macrophage pyroptosis in ALI/ARDS have been increasingly investigated in recent studies, the specific mechanisms of macrophage pyroptosis remain unclear. Whether additional inflammasome complexes or gasdermin proteins revolves in cell pyroptosis remains an open question. At the same time, because of complexity of drugs’ action mechanism, their absorption and distribution in the body are diverse. Therefore, how to select an optimal delivery method for inhibitors to maximize their suppression of macrophage pyroptosis presents a significant challenge. The advent of nanotechnology has opened new avenues for the development of novel therapeutic strategies for the treatment of ARDS/ALI that can utilize targeting macrophage pyroptosis pathways (178). However, its clinical transformation still faces a series of challenges (179).

At the same time, it has not been thoroughly studied whether the potential side effects of inhibitors on the liver, kidney and other organs will aggravate the multiple organs dysfunction of ALI/ARDS. There are few successful examples of macrophage pyroptosis interventions successfully applied in clinical practice, and the application of therapies that inhibit excessive macrophage pyroptosis in clinical practice is still facing severe challenges, while how to clarify the changes of macrophage pyroptosis markers in ALI/ARDS patients is also a key point of carrying out clinical interventions targeting the modulation of pulmonary macrophage pyroptosis. At present, there are few clinical studies related to the treatment of ALI/ARDS patients by modulating macrophage pyroptosis, and more preclinical studies are still needed to confirm the potential beneficial role of regulating macrophage pyroptosis in ALI/ARDS. Some studies have confirmed the role of markers related to pyroptosis in early identification and prognosis assessment of ARDS patients. For example, a study demonstrated that the mean levels of caspase-1, IL-1β and IL-18 in aspirates from ARDS patients were significantly elevated compared to those in healthy individuals (93). Modulation of macrophage pyroptosis-related indicators may become a potential therapeutic approach for ARDS patients, but future clinical studies are still needed to demonstrate the beneficial effects of interventions to modulate macrophage pyroptosis in ARDS patients.

## 7 Conclusion and outlook

As an acute diffuse lung injury occurring in a short period of time, ALI/ARDS has significantly severe clinical prognosis, and therefore its clinical treatment is full of challenges. Macrophage pyroptosis, as a special way of cell death, has attracted much attention in study of development and treatment of ALI/ARDS. Up to now, many studies have shown that macrophage pyroptosis could be affected by regulating mitochondrial function (107, 180), adjusting the generation of exosomes (181) or neutrophil extracellular traps (93), and controlling its upstream signaling transduction (100, 149, 182), thus influencing development and outcome of ALI/ARDS. The regulation of macrophage pyroptosis may become a therapeutic target for ALI/ARDS, thus providing more new research directions for the treatment of ALI/ARDS.

However, there are still some problems unsolved. Firstly, the current understanding of the inflammasomes and their role in pyroptosis is incomplete, with only a few inflammasomes and their mechanisms of action in pyroptosis being well understood. Secondly, most of the current research on macrophage pyroptosis is limited to preclinical studies, and the clinical studies that have been conducted also have problems such as small sample sizes. In clinical practice, there are very few cases where the actual reduction of lung injury through intervention in macrophage pyroptosis has been achieved, making it difficult to prove the clinical utility. Therefore, more and more in-depth basic and clinical trials are needed to investigate the specific role of macrophage pyroptosis in ALI/ARDS, with the aim of selecting more effective targets for the treatment of ALI/ARDS.
